# Predicting Differences in Treatment Response and Survival Time of Lung Adenocarcinoma Patients Based on a Prognostic Risk Model of Glycolysis-Related Genes

**DOI:** 10.3389/fgene.2022.828543

**Published:** 2022-05-25

**Authors:** Rongchang Zhao, Dan Ding, Yan Ding, Rongbo Han, Xiujuan Wang, Chunrong Zhu

**Affiliations:** ^1^ Department of Oncology, Taixing People’s Hospital Affiliated to Bengbu Medical College, Taixing, China; ^2^ Department of Intensive Care Unit, Taixing People’s Hospital Affiliated to Bengbu Medical College, Taixing, China; ^3^ Department of Oncology, The First Affiliated Hospital of Soochow University, Suzhou, China

**Keywords:** lung adenocarcinoma, glycolysis, nomogram, drug sensitivity, tumor mutation burden

## Abstract

**Background:** Multiple factors influence the survival of patients with lung adenocarcinoma (LUAD). Specifically, the therapeutic outcomes of treatments and the probability of recurrence of the disease differ among patients with the same stage of LUAD. Therefore, effective prognostic predictors need to be identified.

**Methods:** Based on the tumor mutation burden (TMB) data obtained from The Cancer Genome Atlas (TCGA) database, LUAD patients were divided into high and low TMB groups, and differentially expressed glycolysis-related genes between the two groups were screened. The least absolute shrinkage and selection operator (LASSO) and Cox regression were used to obtain a prognostic model. A receiver operating characteristic (ROC) curve and a calibration curve were generated to evaluate the nomogram that was constructed based on clinicopathological characteristics and the risk score. Two data sets (GSE68465 and GSE11969) from the Gene Expression Omnibus (GEO) were used to verify the prognostic performance of the gene. Furthermore, differences in immune cell distribution, immune-related molecules, and drug susceptibility were assessed for their relationship with the risk score.

**Results:** We constructed a 5-gene signature (FKBP4, HMMR, B4GALT1, SLC2A1, STC1) capable of dividing patients into two risk groups. There was a significant difference in overall survival (OS) times between the high-risk group and the low-risk group (*p* < 0.001), with the low-risk group having a better survival outcome. Through multivariate Cox analysis, the risk score was confirmed to be an independent prognostic factor (HR = 2.709, 95% CI = 1.981–3.705, *p* < 0.001), and the ROC curve and nomogram exhibited accurate prediction performance. Validation of the data obtained in the GEO database yielded similar results. Furthermore, there were significant differences in sensitivity to immunotherapy, cisplatin, paclitaxel, gemcitabine, docetaxel, gefitinib, and erlotinib between the low-risk and high-risk groups.

**Conclusion:** Our results reveal that glycolysis-related genes are feasible predictors of survival and the treatment response of patients with LUAD.

## Introduction

Lung cancer accounts for a large proportion of cancer-related human deaths worldwide (Barta et al., 2019; Bade and Dela Cruz, 2020). Because lung adenocarcinoma (LUAD) is a common pathological type of lung cancer (Travis et al., 2015), individualized therapy for LUAD has received increasing attention from clinicians. Because tumor occurrence and development are based on genetic changes (2012; Vogelstein et al., 2013; Zhu et al., 2015), response to therapies and overall survival (OS) is not necessarily the same in patients of the same gender, performance status score, age, and TNM stage when the influence of social, family, and economic factors is removed. Therefore, there is an urgent need to explore effective microscopic molecular biomarkers to predict the response and prognosis of LUAD patients.

Understanding the differences in metabolism and proliferation between tumor cells and normal cells is essential to predict the prognosis and clinical response to treatment in cancer patients. Cells mainly obtain energy to perform their biological activities through glycometabolism, and LUAD cells are not an exception to this rule. Previous studies have revealed that the most significant metabolic change in cancer cells is the appearance of the Warburg effect, which is manifested by increased aerobic glycolysis of tumor cells and dependence on glycolytic pathways to produce adenosine triphosphate (ATP) (Koppenol et al., 2011; Xu et al., 2015; Schwartz et al., 2017; Vaupel et al., 2019). In view of this unique metabolic alteration in tumor cells, many targeted treatments have been discovered and have improved over time (Danial et al., 2003; Coy et al., 2005; Xu et al., 2005; Pelicano et al., 2006; Yang et al., 2020). In addition, an increasing number of studies have used glycolysis-related genes to establish predictive models of tumor prognosis (Zhang et al., 2019; Liu et al., 2020; Tang et al., 2020; Wu et al., 2020; Zhou et al., 2020). The tumor mutation burden (TMB) is defined as the number of somatic mutations per megabase (Mb) of the genomic sequence interrogated of a tumor and can be used as a predictor of the efficacy of immune checkpoint inhibitors (ICI) in multiple tumors (Snyder et al., 2014; Rizvi et al., 2015; Dong et al., 2017). In recent years, several studies have evaluated the TMB as a prognostic molecular marker (Kang K. et al., 2020; Lv et al., 2020; Zhang et al., 2020). Since glycolysis-related genes can be used to establish an effective prognosis prediction model for LUAD, integrating TMB into the prediction model is a new and promising approach with inherent advantages.

Here, we conducted in-depth study using gene expression data from patients with LUAD based on data extracted from TCGA database and used differences in TMB to screen glycolysis-related genes. Subsequently, we used statistical tools to obtain a prognostic model and nomogram composed of glycolysis-related genes, which exhibited good clinical applicability and produced a method for reliable prediction of prognosis. Furthermore, the risk score was associated with the tumor immune microenvironment and could be used to estimate the sensitivity of the LUAD patients included in this study to treatments.

## Materials and Methods

### Clinical Information and Gene Expression Data From Patients

Clinical characteristics, genetic mutations, and mRNA expression data from patients with LUAD were extracted from TCGA database (https://cancergenome.nih.gov/) for subsequent analyzes using Perl (version 5.32.1.1). The clinical characteristics of 522 patients with LUAD, including gender, age, T (tumor), N (lymph node), and M (metastasis) stages, clinical stage, survival status, and smoking history, are detailed in Table 1. We defined smoking history as: a lifelong nonsmoker (<100 cigarettes smoked in lifetime) = 1, current smoker (includes daily smokers and non-daily smokers or occasional smokers) = 2, current reformed smoker for >15 years = 3, current reformed smoker for ≤15 years = 4, current reformed smoker, duration not specified = 5).

**TABLE 1:** LUAD patient characteristics in the TCGA.

Clinical feature	N	%
Gender		
Male	242	46.4
Female	280	53.6
Age (years)		
≤65	241	46.2
>65	262	50.2
Unknown	19	3.6
Stage		
Stage I	279	53.4
Stage II	124	23.8
Stage III	85	16.3
Stage IV	26	5.0
Unknown	8	1.5
T (tumour)		
T1	172	33.0
T2	281	53.8
T3	47	9.0
T4	19	3.6
TX	3	0.6
N (lymph node)		
N0	335	64.2
N1	98	18.8
N2	75	14.4
N3	2	0.4
NX	11	2.0
Unknown	1	0.2
M (metastasis)		
M0	353	67.6
M1	25	4.8
MX	140	26.8
Unknown	4	0.8
Smoking history		
1	75	14.4
2	122	23.3
3	137	26.2
4	170	32.6
5	4	0.8
Unknown	14	2.7
Survival status		
Death	188	36.0
Alive	334	64.0

### Screening of Differentially Expressed Glycolysis-Related Genes

By searching glycolysis gene sets in the Gene Set Enrichment Analysis (GSEA) database (http://www.broadinstitute.org/gsea/index.jsp), a total of 326 genes were obtained. The gene mutation data obtained were used to calculate the TMB value of each LUAD sample and were divided into high and low TMB groups based on the median value. Subsequently, the analysis of differentially expressed glycolysis-related genes between the high TMB group and the low TMB group was performed in R, with the following cut-off criteria: |log2fold change (logFC)| > 0.2; *p*-value < 0.05; FDR (false discovery rate) < 0.05.

### Gene Prognosis Model Development and Expression Validation

The ‘survival’ and ‘glmnet’ packages in R were used to perform the univariate Cox regression analysis of differentially expressed genes. LASSO regression analysis and multivariate Cox proportional hazards regression were performed. We use the following risk formula: Risk score = β gene1 × Expressiongene1 + β gene2 × Expressiongene2 + β gene3 × Expressiongene3 + + β genen × Expressiongenen, where β is the coefficient and Expressiongene indicates the expression level in patients with LUAD. LASSO is a popular machine-learning algorithm, which was extensively utilized in medical studies (Liu et al., 2021; Liu et al., 2022a; Liu et al., 2022b; Liu et al., 2022c). ROC curves were used to evaluate the validity of the prognostic model using the ‘survival ROC’ package. Differences in survival times between the low-risk and high-risk groups was then assessed using the ‘survival’ package and the ‘survminer’ package in R, and Kaplan–Meier (KM) curves were plotted to display the results directly. Finally, by integrating GSE68465 and GSE11969 datasets, 536 LUAD patient data points were obtained, and patients were divided into high- and low-risk groups using the risk score formula. The Human Protein Atlas (HPA) database (https://www.proteinatlas.org/) was used to verify the expression of genes in the model in LUAD cells.

### Comparison of Prognostic Model Prediction Performance

The gene prognostic model was compared with gender, age, stage T (tumor), stage N (lymph node), M (metastasis), clinical stage, and smoking history as an independent prognostic analysis. Subsequently, the AUC of the model was compared with that of the clinicopathological features and with that of the existing LUAD prognostic model with a similar number of genes.

### Prognostic Performance of Risk Genes and Nomogram Construction

The prognostic value of the genes included in the model was verified using the Gene Expression Profiling Interactive Analysis (GEPIA) database (http://gepia.cancer-pku.cn/) and the Kaplan-Meier (KM) plotter database (http://kmplot.com/analysis/). The results of differences in clinicopathological characteristics between the high-risk and low-risk groups were displayed in a strip chart. The differences in risk scores each clinicopathological feature are presented in the box plot. Where *p* < 0.001 = * **, *p* < 0.01 = ** *, *p* < 0.05 = *. Furthermore, we built a nomogram to improve the application of the model in the clinical setting and validated it using the ‘rms’ package. A nomogram is a prognostic evaluation tool that can integrate several prognostic determinants, including molecular and clinicopathological parameters; and can calculate and visualize the numerical probability of clinical events using a relatively simple output and is a tool widely used in clinical oncology (Balachandran et al., 2015).

### Immune Microenvironment and Therapeutic Response

To clarify the relationship between the immune microenvironment and the risk score, XCELL, TIMER, QUANTISEQ, MCPcounter, EPIC, CIBERSORT, and CIBERSORT-ABS were used to obtain differences in the distribution of immune cells in patients with LUAD from TCGA database. The expression of genes related to immune checkpoint inhibitors were compared between the low-risk group and the high-risk group. We showed the results and the *p*-value was labeled as follows: *p* < 0.001 = ***, *p* < 0.01 = ***, and *p* < 0.05 = *. The Tumor Immune Dysfunction and Exclusion (TIDE) database (http://tide.dfci.harvard.edu) was used to obtain scores of immunotherapy evaluation-related indicators of LUAD samples (Hoshida et al., 2007; Jiang et al., 2018). Meanwhile, a differential comparative analysis was performed using the number of responder and non-responder patients evaluated after immunotherapy obtained from the website. Subsequently, we added the pRRophetic package to evaluate the differences in clinical responses between LUAD patients grouped by the gene prognostic model by calculating the IC50, or half maximal inhibitory concentration, of commonly used drugs in R.

### Statistical Analysis

All statistical analyses were performed using R 4.1.2 (https://www.r-project.org/). Univariate Cox regression analysis was used to screen for genes associated with prognosis with *p* < 0.0001. Multivariate Cox proportional hazards regression and LASSO regression analysis were used to construct prognostic model. The KM survival curve analysis and a log-rank test were used to compare differences in overall survival time between the high-risk group and low-risk group. The relationship between risk score and clinicopathological features was analyzed using The Chi-Square test and Wilcoxon Signed-Rank Test with *p* < 0.05. Spearman’s correlation analysis was performed to obtain the correlation between the distribution of immune cell and risk score. Differences between immune cells in risk groupings were analyzed using the Wilcoxon signed rank test with *p* < 0.0001. The Wilcoxon test was used to calculate the TIDE score and the T cell exhaustion potential of the tumor score between the high- and low-risk groups. Chi-square test was used to obtain *p*-values to compare the differences in the number of immune responses in different groups.

## Results

### Data Processed by GSEA

**FIGURE 1:**
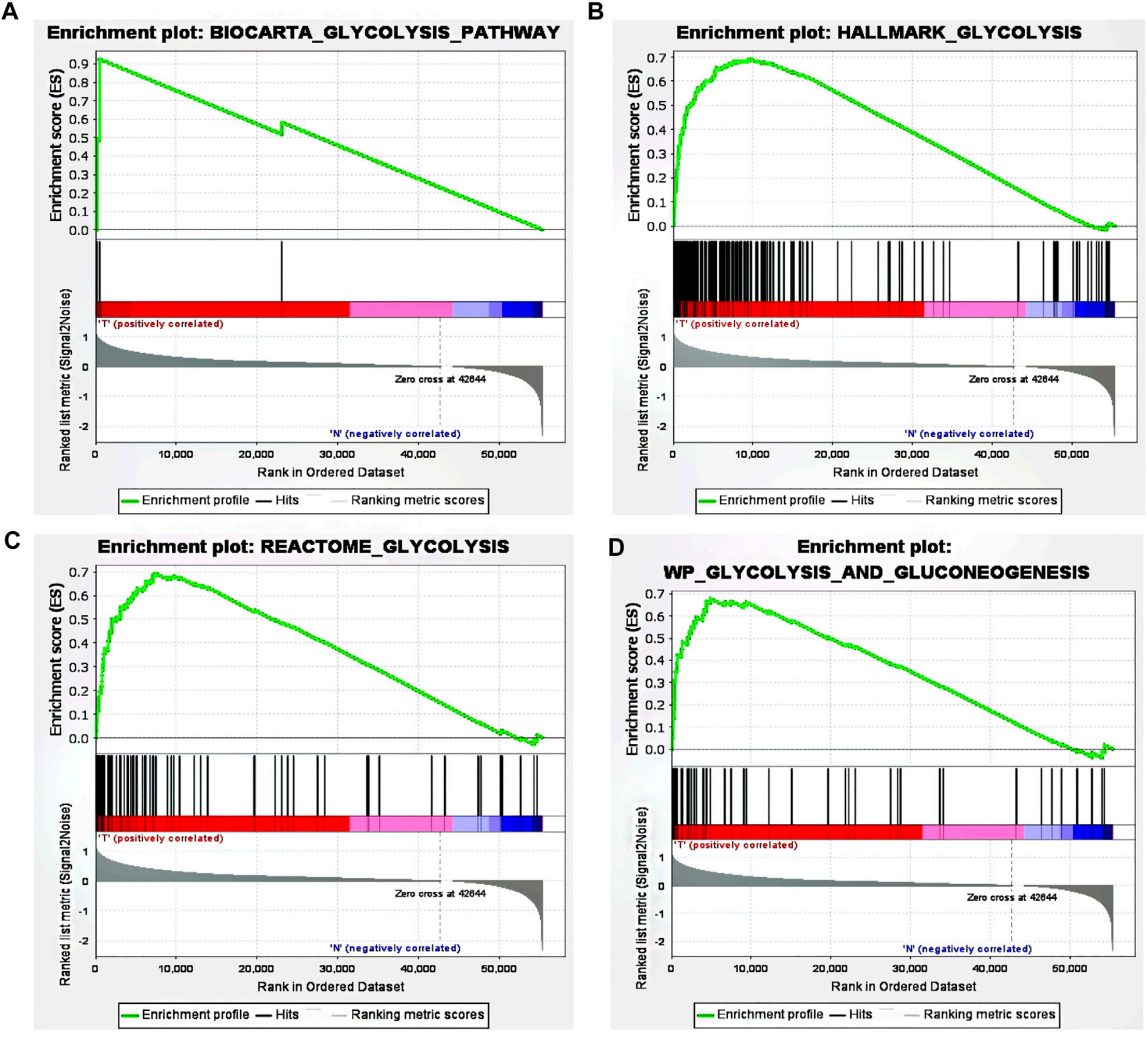
GSEA revealed that four gene sets were significantly enriched: **(A)** BioCarta glycolysis **(B)** glycolysis **(C)** REACTOME glycolysis, and **(D)** WP glycolysis and gluconeogenesis.

Using ‘glycolysis’ as the search keyword, the BioCarta, Hallmark, KEGG, REACTOME and WP gene sets were selected from the Molecular Signatures Database (MSigDB) to obtain a glycolysis-related gene signature. After using the above detailed data to perform GSEA, significant differences were detected between the tumor tissue sample group and the normal tissue sample group among the gene sets (Figures 1A–D), with normalized *p* values <0.05.

### Establishment and Evaluation of a Glycolysis-Related Gene Model

**FIGURE 2:**
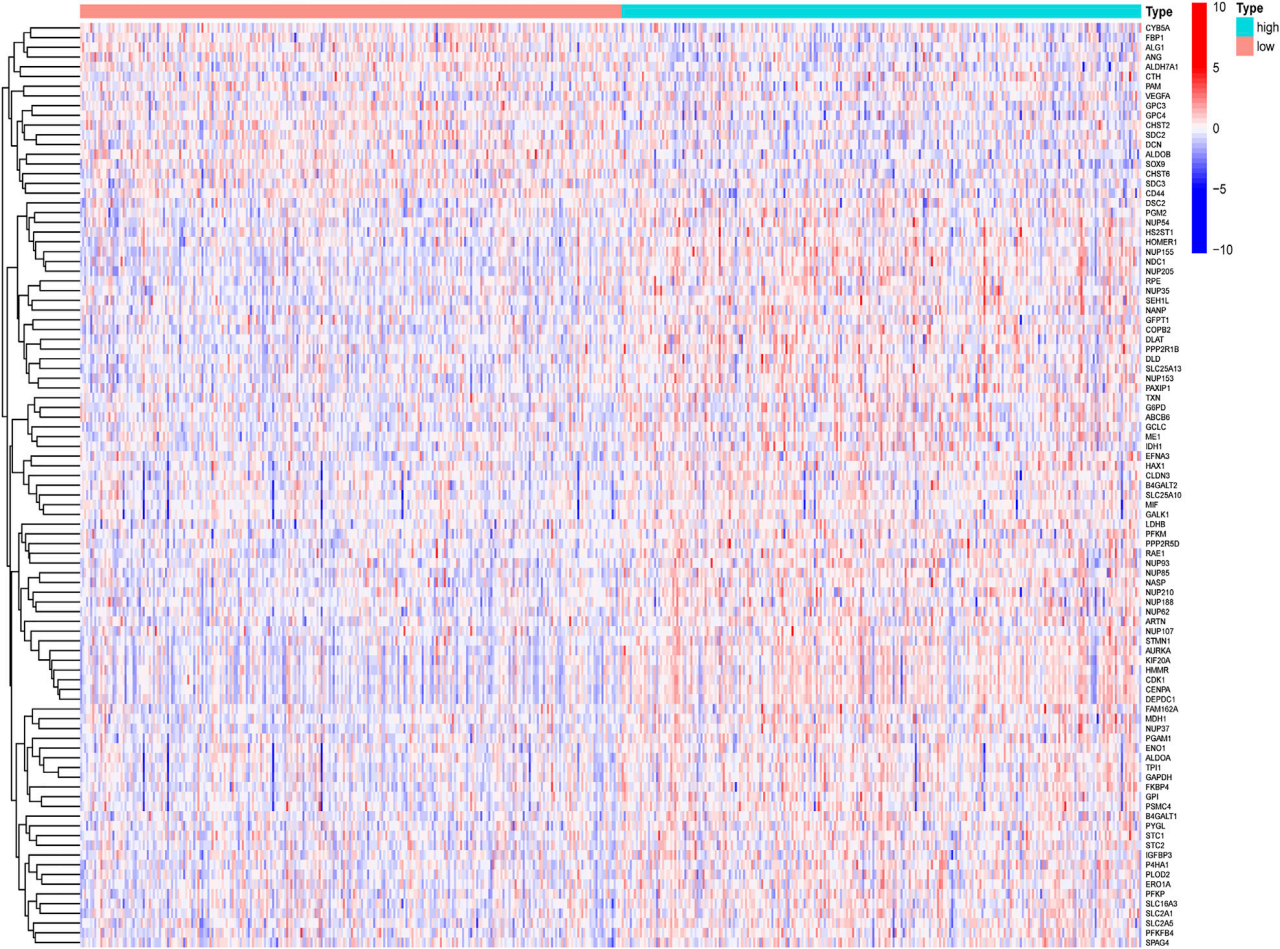
Heatmap diagram of differentially expressed glycolysis-related genes, by comparing the high TMB group with the low TMB group.

**FIGURE 3:**
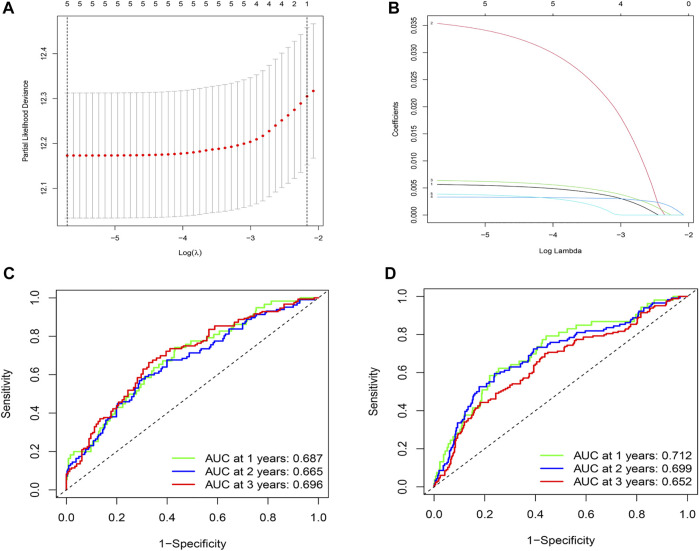
Construction and evaluation of prognostic models. **(A)**The LASSO coefficient was calculated. **(B)** The partial likelihood deviance of the LASSO coefficient was calculated. **(C)** AUC values of 1-, 2-, and 3-years survival rates are shown in the ROC curve, by integrating TCGA database data. **(D)** The AUC values of 1-, 2-, and 3-years survival rates are shown in the ROC curve, by integrating the data from the GEO database.

**FIGURE 4:**
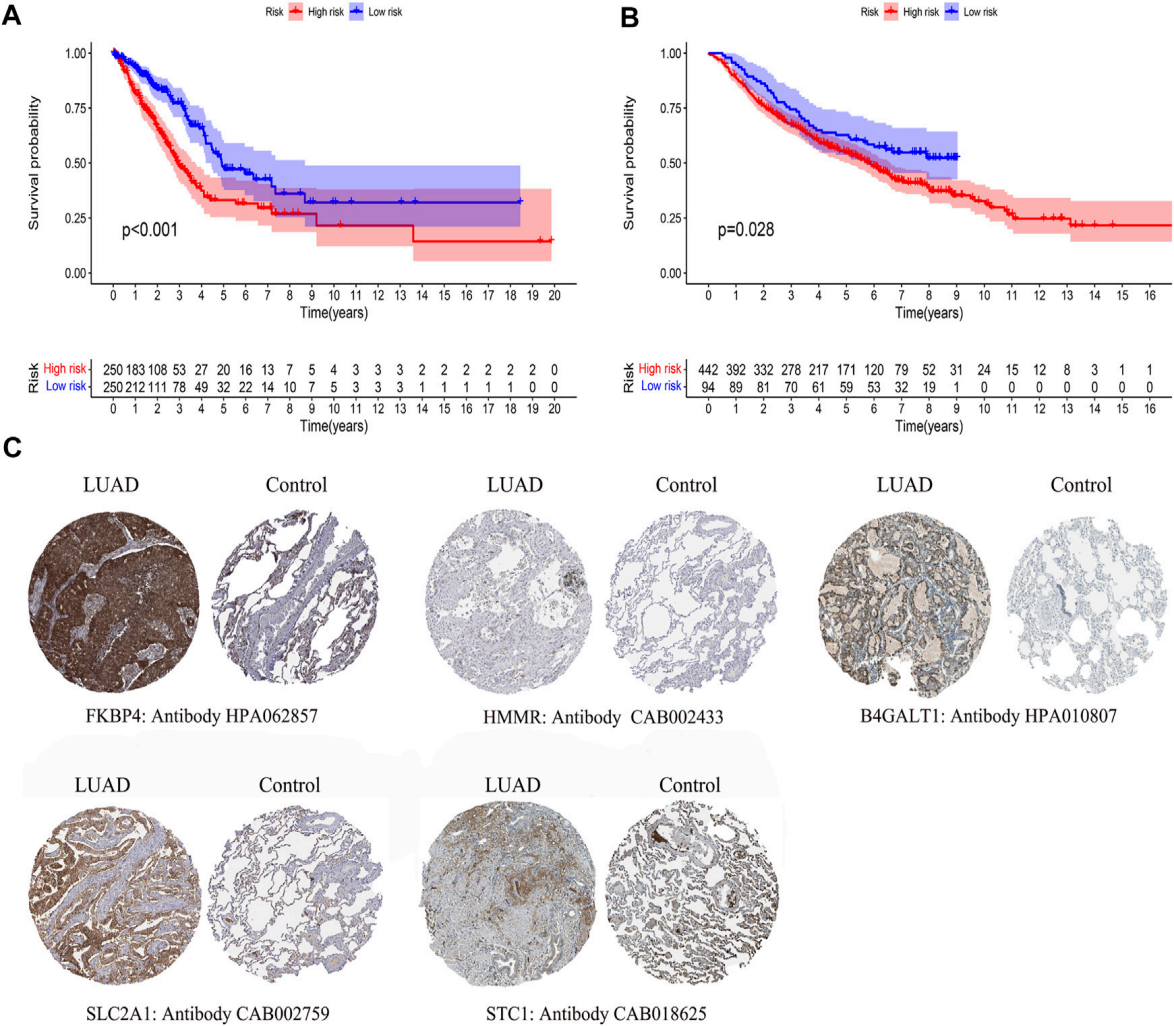
Differences in survival by model grouping and differences in expression of model genes. **(A)** Kaplan-Meier survival curves of patients based on the risk score of 5 glycolysis-related genes, from TCGA database. **(B)** Kaplan-Meier survival curves of patients based on the risk score of 5 glycolysis-related genes, from the GEO database. **(C)**Immunohistochemical results of LUAD and normal lung tissue, including FKBP4, HMMR, B4GALT1, SLC2A1, STC1.

**FIGURE 5:**
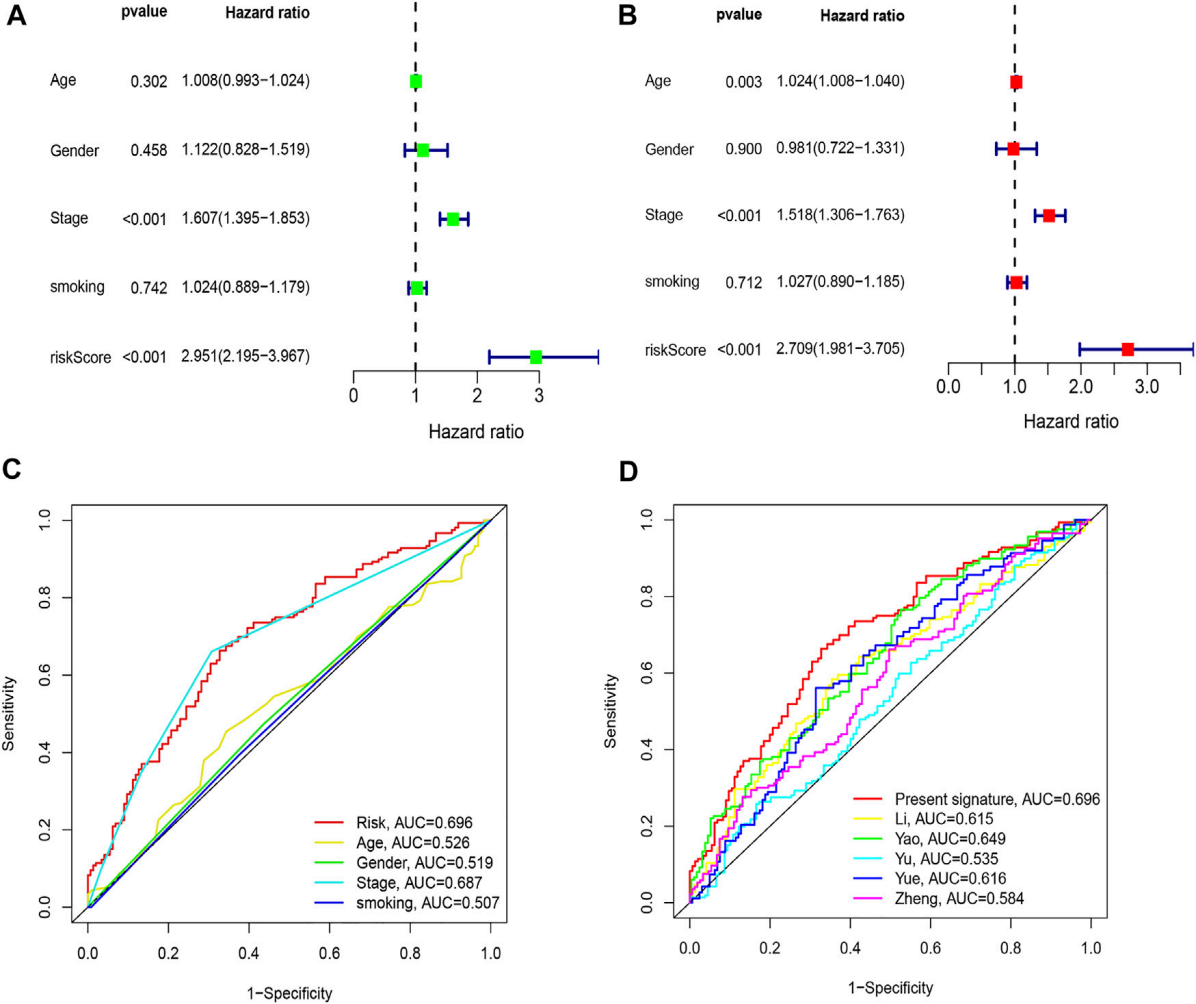
Comparison of clinicopathologic features with other model. **(A)** Univariate Cox regression analyses. **(B)** Multivariate Cox regression analyses. **(C)** AUC of the risk score, age, gender, and stage. **(D)** AUC of the similar prognostic models.

**FIGURE 6:**
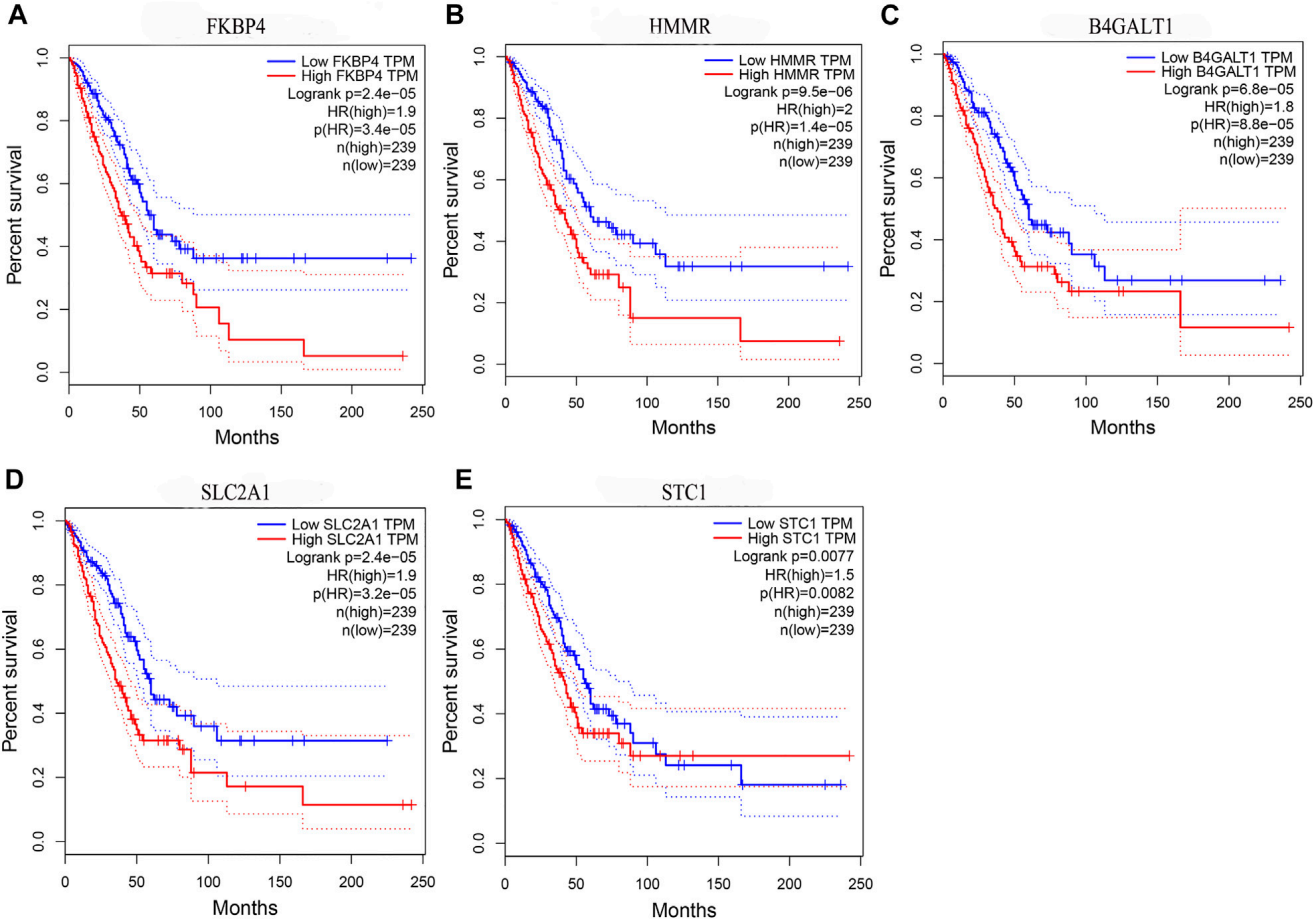
Prognostic validation of risk genes. **(A)** FKBP4 **(B)** HMMR **(C)** B4GALT1 **(D)** SLC2A1, and **(E)** STC1 all have the ability to predict survival time, in the GEPIA database.

**FIGURE 7:**
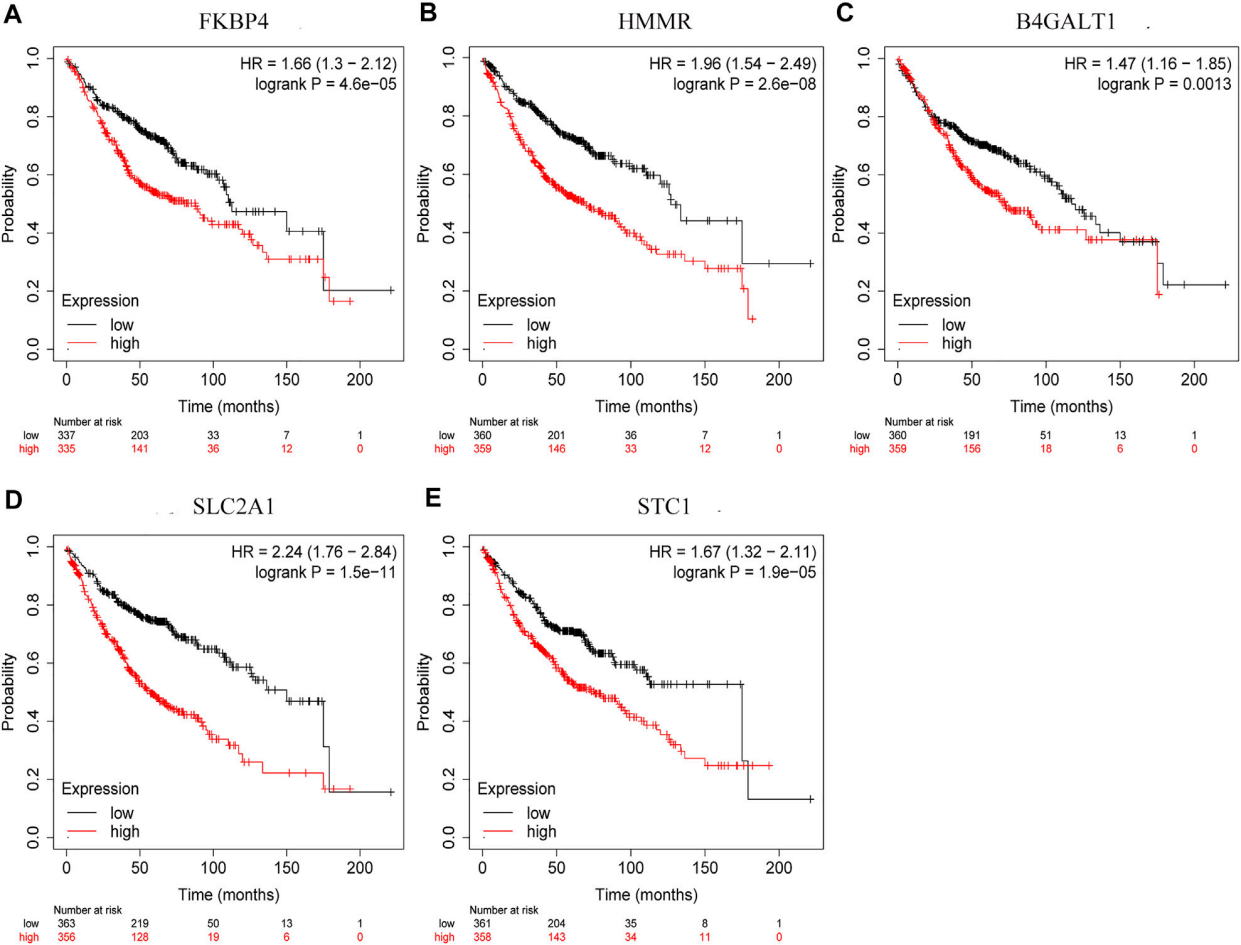
Prognostic validation of risk genes. **(A)** FKBP4 **(B)** HMMR **(C)** B4GALT1 **(D)** SLC2A1, and **(E)** STC1 also have the ability to predict survival time, in Kaplan-Meier (KM) plotter database.

By comparing the high TMB group with the low TMB group, 95 differentially expressed genes were obtained (Figure 2 and Supplementary Material 1). Univariate Cox analysis was used obtain 10 genes related to prognosis (Table 2). LASSO regression analysis and multivariate Cox proportional hazards regression analysis were used to calculate the risk score to construct the prognostic model (Figures 3A,B and Supplementary Table S1). Prognosis was estimated as follows: (0.00565 * expression level of FKBP4) + (0.03539 * expression level of HMMR) + (0.00638 * expression level of B4GALT1) + (0.00332 * SLC2A1) + (0.00387 * expression level of STC1). The areas under the ROCs (AUCs) of the 1-, 2-, and 3-years overall survival (OS) rate analysis for the 5-gene prognostic model were 0.687, 0.665 and 0.696, respectively, (Figure 3C). Similar verification results were obtained in the GEO database, and the ROCs (AUCs) of the 1-, 2-, and 3-years OS rate analysis for the prognostic model were 0.712, 0.699 and 0.652, respectively, (Figure 3D). Survival was significantly different between the two groups (*p* < 0.001), in TCGA database (Figure 4C). The low-risk group in the GEO database also had a better prognosis (*p* = 0.028) (Figure 4B). Immunohistochemical results of FKBP4, HMMR, B4GALT1, SLC2A1 and STC1 gene expression status in LUAD tissue and normal tissue obtained from HPA database are shown in Figure 4C. We conducted an independent prognostic analysis of age [*p* = 0.302, HR = 1.008, 95% CI (0.993–1.024)], gender [*p* = 0.458, HR = 1.122, 95% CI (0.828–1.519)], stage [*p* < 0.001, HR = 1.607, 95% CI (1.395–1.853)], smoking [*p* = 0.742, HR = 1.024, 95% CI (0.889–1.179)] and risk score [*p* < 0.001, HR = 2.951, 95% CI (2.195–3.967)] by univariate Cox regression analysis (Figure5A) and confirmed that stage [*p* < 0.001, HR = 1.518, 95% CI (1.306–1.763)] and risk score [*p* < 0.001, HR = 2.709, 95% CI (1.981–3.705)] can be used as predictors of prognosis in LUAD patients by multivariate Cox regression analysis (Figure 5B). We also found that the AUC of the risk score of all patients was similar to the AUC of the clinical stage (Figure 5C). The predictive performance of the prognostic model in this paper is significantly better than the results in other studies in the 3-years survival time (Figure 5D) (Yue et al., 2019; Li J. et al., 2020; Yu and Tian, 2020; Yao et al., 2021; Zheng et al., 2021). Validation in the GEPIA database suggests that each gene in the model has a prognostic value (Figures 6A–E), and similar results were also obtained in the Kaplan–Meier (KM) plotter database (Figures 7A–E).

**TABLE 2:** Univariate Cox regression analysis.

ID	HR	95% CI		*p* value
		Lower	Upper	
SLC16A3	1.021	1.014	1.028	2.80e–08
FKBP4	1.011	1.006	1.015	1.29e–05
GAPDH	1.001	1.000	1.001	4.30e–08
HMMR	1.062	1.031	1.092	4.55e–05
B4GALT1	1.009	1.006	1.013	5.39e–07
SLC2A1	1.006	1.004	1.009	9.44e–08
PGAM1	1.039	1.020	1.059	7.24e–05
PGM2	1.053	1.029	1.078	1.12e–05
STC1	1.009	1.004	1.013	7.94e–05
ERO1A	1.013	1.008	1.017	2.61e–08

### Clinical Evaluation and Nomogram Construction

**FIGURE 8:**
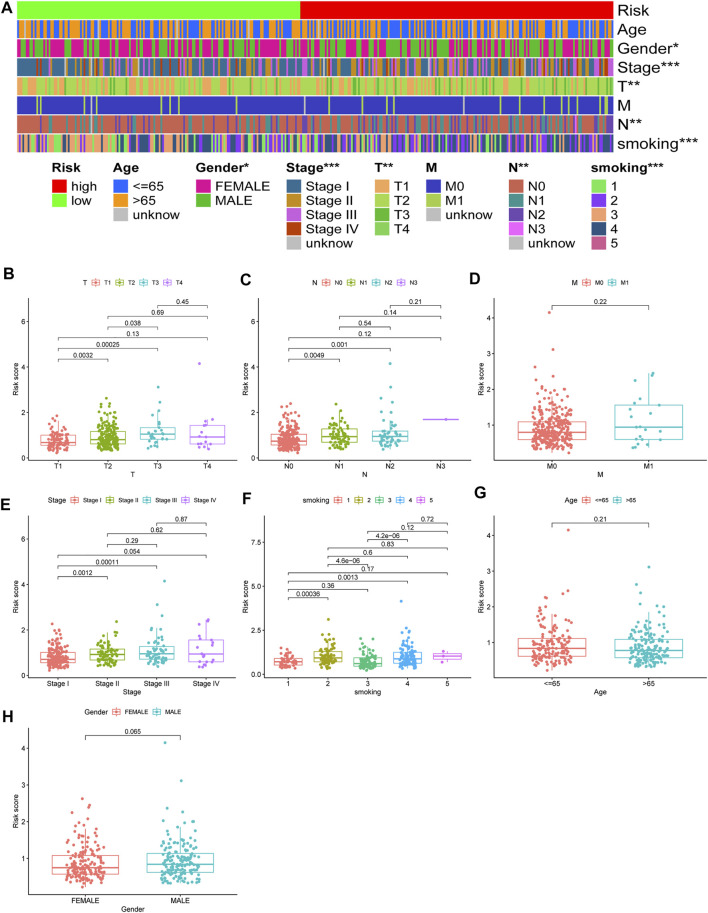
Use of risk assessment models for clinical evaluation. **(A)** A strip chart for an overview of information. The scatter diagram of **(B)** T stage **(C)** N stage **(D)** M stage **(E)** clinical stage **(F)** smoking **(G)** age, and **(H)** gender.

**FIGURE 9:**
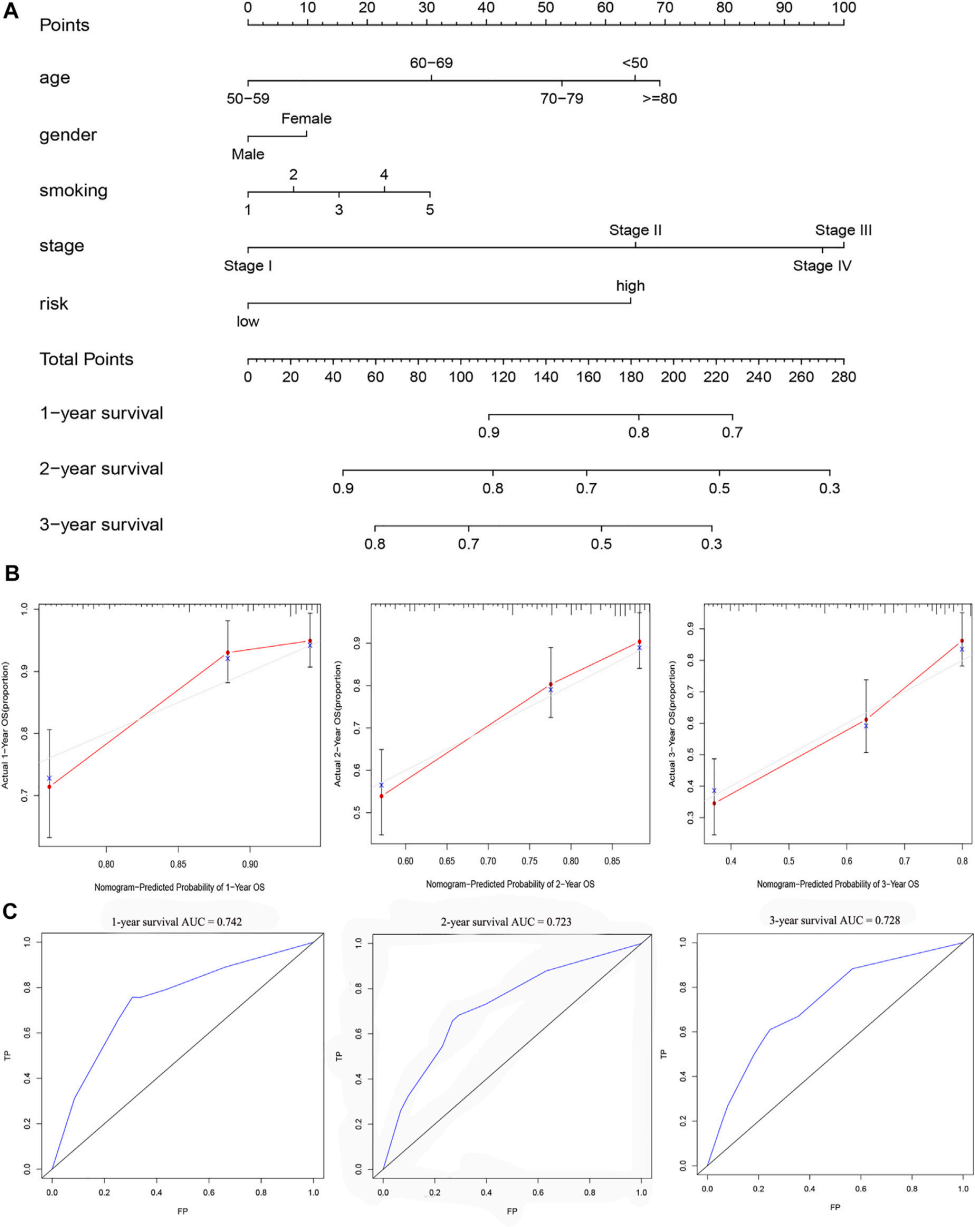
A nomogram for survival prediction was constructed and evaluated. **(A)** The nomogram to predict the 1-, 2- and 3-years survival rate of LUAD patients. **(B)**The consistency of the actual proposal and the predicted probability of OS according to the nomogram at 1, 2 and 3 years. **(C)**AUC calculated for 1 year, 2 years and 3 years based on the nomogram.

Chi-square test results suggested statistically significant differences in gender, T stage, N stage, clinical stage, and smoking status between subgroups of prognostic models (Figure 8A). We then found that T stage, N stage, clinical stage, and smoking status (Figures 8B,C,E,F) were significantly correlated with the risk score, while there were no differences in risk scores between the M stage, age, and gender group (Figures 8D,G,H) using the Wilcoxon signed-rank test. Subsequently, to enhance the clinical utility of the gene model, we constructed a nomogram (Figure 9A). The risk score, age, gender, smoking status, and clinical stage were basic elements included in the nomogram. Furthermore, the nomogram prediction results were generally consistent with the actual survival outcomes based on the prediction calibration curves of the 1-, 2-, and 3-years survival rates (Figure 9B). The AUCs of the 1-, 2-, and 3-years survival rate predictions for the nomogram were 0.742, 0.723, and 0.728 (Figure 9C).

### Estimation of Tumor Immune-Related Cells and Molecules and Patient Therapeutic Response With the Gene Assessment Model

**FIGURE 10:**
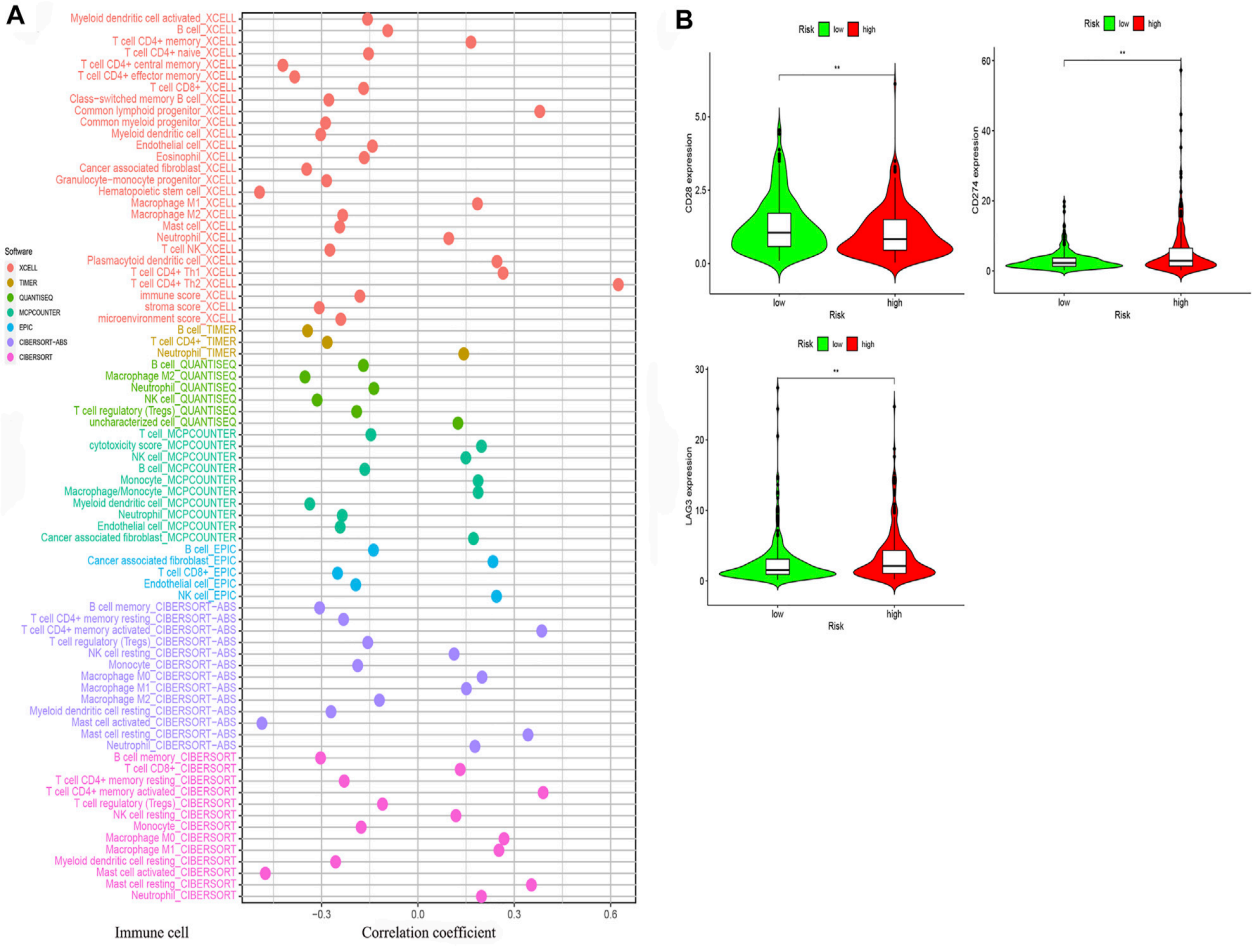
The risk model was used to evaluate tumor-infiltrating cells and immune costimulatory molecules. **(A)** Correlation between immune cells and the risk score was obtained using different methods. **(B)** A low-risk score was positively related to upregulation of CD28, CD274, and LAG3 expression.

**FIGURE 11:**
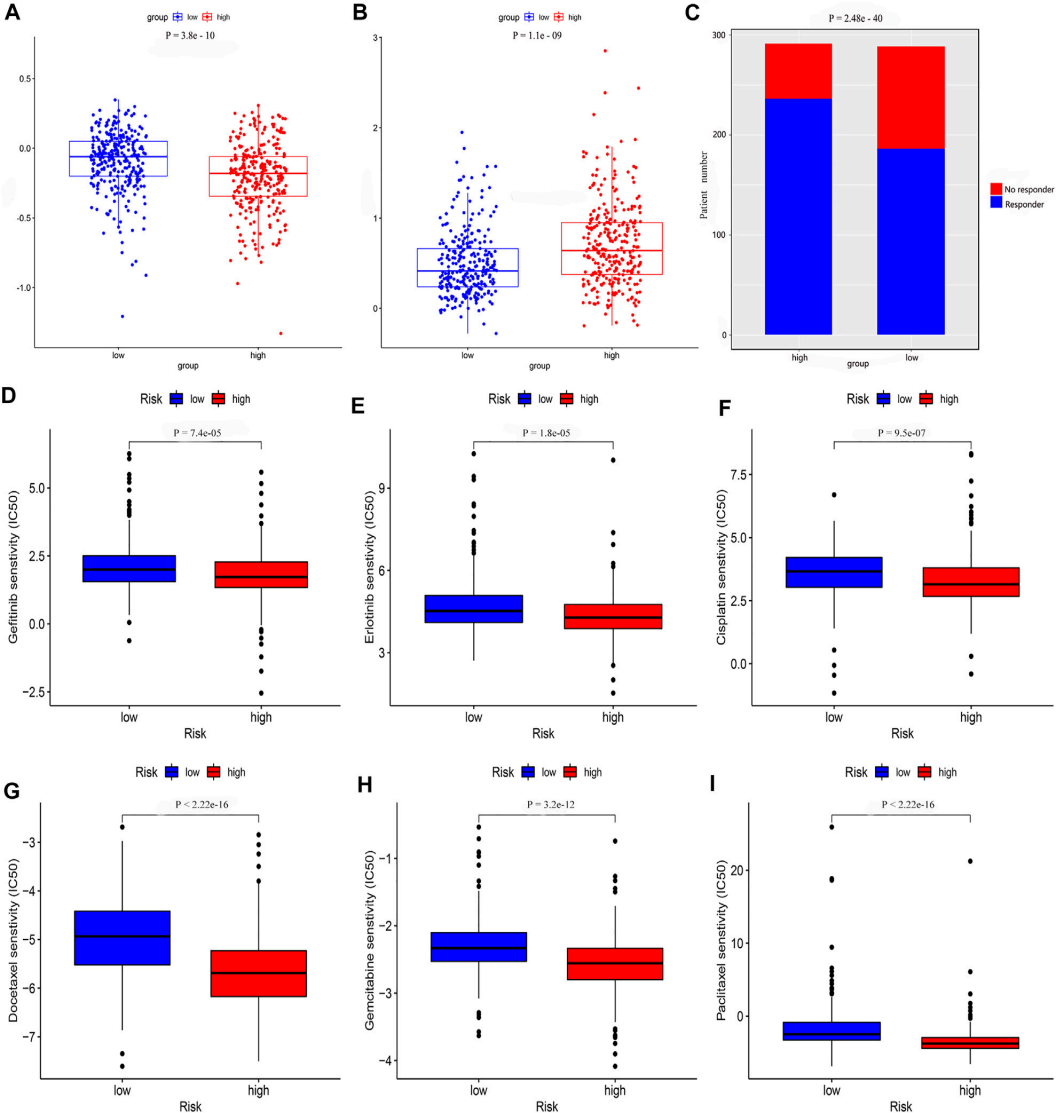
The risk model was used to evaluate drug treatment differences. Analysis of **(A)** TIDE value **(B)** T cell exhaustion potential of the tumor score **(C)** Number of no responder and responder patients to immune checkpoint inhibitors **(D)** gefitinib **(E)** erlotinib **(F)** cisplatin **(G)** docetaxel **(H)** gemcitabine, and **(I)** paclitaxel showed that there was a significant difference between the low- and high-risk groups.

We determined that the presence of tumor immune-related cells, such as myeloid dendritic cells, CD4^+^ memory T cells, CD8^+^ T cells, endothelial cells, and M2 macrophage, was positively correlated with low risk, while common lymphoid progenitor, M0 macrophages, M1 macrophages, and NK cells were positively associated with high risk (Figure 10A). Detailed results of the Wilcoxon-signed rank test are shown in Supplementary Material 2. Since it is not uncommon to use immune checkpoint inhibitors to treat LUAD in the clinic, our aim was to clarify whether ICI-related biomarkers were associated with the risk model and found that low risk scores were positively correlated with high expression of CD28 (*p* < 0.01), CD274 (*p* < 0.01), and LAG3 (*p* < 0.01) (Figure 10B). Subsequently, we found in the TIDE database that the TIDE score of the low-risk group was higher than that of the high-risk group (*p* = 3.8e–10) (Figure 11A). This suggested that the possibility of immune escape in the low-risk group is higher than in the high-risk group, and the immunotherapy effect is worse than that in the high-risk group. The T cell exhaustion potential of the tumor score was higher in the high-risk group than in the low-risk group (Figure 11B). Combined analysis of TIDE value and IFNG gene expression indicated that the number of effective samples for immune checkpoint blockade in the high-risk group was significantly higher than in the low-risk group (*p* = 2.48e–40) (Figure 11C). Next, we explored the relationship between the risk score and the response to chemotherapy in patients with TCGA LUAD. Our results indicated that a low-risk score was correlated with a higher IC50 for medications such as gefitinib (*p* = 7.4e–05), erlotinib (*p* = 1.8e–05), cisplatin (*p* = 9.5e–07), docetaxel (*p* < 2.22e–16), gemcitabine (*p* = 3.2e–12), and paclitaxel (*p* < 2.22e–16) (Figures 11D–I).

## Discussion

Although survival rate and quality of life of patients with LUAD has improved with the development of multiple aggressive treatments, such as surgery, chemotherapy, targeted therapy, and radiotherapy; not every patient can benefit from these treatments. The reason for this phenomenon is that most of the genes altered in different lung adenocarcinoma patients are different. Therefore, existing guidelines recommend genetic testing for lung adenocarcinoma patients before treatment, such as EGFR mutations and anaplastic lymphoma kinase (ALK) fusion mutations (Zhu et al., 2008; Shan et al., 2015; Khan et al., 2018; Kang J. et al., 2020). However, there are still some patients with the same expression of the above-mentioned genes with different prognosis. For example, only some of these patients effectively respond to immune checkpoint inhibition therapy, while others still progress rapidly (Herbst et al., 2020). Thus, we should conduct in-depth research and discussion on the types and number of genes that need to be detected. To date, many studies have revealed that the integration of expression data of multiple molecular markers can effectively predict patient prognosis and their potential response to drugs. Of these, the breast cancer 21-Gene Expression Assay is one of the most well-developed panels; it can provide a prediction of patient prognosis, disease recurrence, and tumor metastasis and can be used to guide treatment plans and assist in the development of individualized patient treatment strategies (Sparano et al., 2018). Based on this research model, the research on molecular markers of lung adenocarcinoma is also in full swing in recent years. However, the research directions of these studies have been different, such as the development of an immune prognostic model (Luo et al., 2020), an autophagy-associated gene prognostic model (Wang X. et al., 2020), a ferroptosis-related gene prognostic model (Gao et al., 2021), and a glycolysis-related gene prognostic model (Liu et al., 2020), so it is not known whether any one approach is effective for all individuals. Therefore, continuously improving predictive model methods will provide a variety of treatment options for specific patients.

To obtain a more reliable prognostic model for LUAD, we used prognostic models constructed from glycolysis-related genes as a reference. First, glycolysis-related genes in LUAD were extracted and, based on differences in the TMB, differentially expressed glycolysis-related genes were selected as the basis to construct the prognostic model. Following Cox regression analyses and LASSO regression analysis, we found that a prognostic model consisting of 5 glycolysis-related genes achieved a better independent prognostic prediction performance, and the nomogram combined with the clinical characteristics of this model resulted in a better performance and more practical clinical application value. Subsequently, we searched the HPA database for the immunohistochemical data relative to these five genes. We also used the data in the GEO database for verification. Since there was a difference in the survival times between patients grouped according to the model, we investigated the reason for this difference. The results of our in-depth study revealed that there were differences in tumor pathological characteristics and immune responses between patients grouped according to glycolysis-related genes, as well as differences in sensitivity to therapeutic drugs. Therefore, we provide sufficient evidence to demonstrate that the gene model obtained in this study contributed to improve the prediction of LUAD patient response to clinical treatment.

For the models obtained in this study, in addition to the predictive performance comparison, we also compared the results of existing similar studies. Sun et al. reported that immune-related genes could be used to construct a prognostic model. However, their nomogram did not combine the model with clinicopathological characteristics, so it was impossible to evaluate the effects of age, gender, and stage for a specific patient (Sun et al., 2020). Although Xu et al. obtained prognostic biomarkers by analyzing the tumor microenvironment of LUAD, they did not calculate the AUC value of the model (Xu et al., 2020). Most risk models are based on detecting the expression level of the molecule of interest and by calculating the total risk score to determine the prognosis of the patient. The first requirement is to judge the accuracy of the model before considering whether it can be used in clinical practice. Li et al. found that RNA binding proteins could be prognostic signatures for LUAD; the obtained model had good prediction performance, and a nomogram was also constructed (Li et al., 2020b). However, the differences in the immune microenvironment between the groups based on that model have not been further explored. It is well known that patient prognosis is related to a variety of factors, and the aforementioned model is of limited use for predicting survival. In addition, the clinical treatment plan for patients is somewhat variable. Therefore, a model is more valuable if it also has the ability to predict the patient’s response to treatment. Wu et al. validated a LUAD prognostic biomarker constructed using autophagy-related long noncoding RNAs (Wu et al., 2021), but the risk model did not specify the 1-, 2-, or 3-years survival rate AUCs in detail, nor did it analyze the relationship with clinicopathological features. Zhang et al. also constructed a prognostic model based on glycolysis-related genes, but did not specify the AUC values for 1, 2, and 3 years, and did not use the TMB in a differential analysis, nor was the model verified (Zhang et al., 2019). The number of genes in the model was greater than that in this study. Our prognostic model, based on the metabolic characteristics of the tumors, has the following favorable characteristics. First, it is supported by a strong theoretical basis. Glycolysis, as a metabolic characteristic of common tumor changes, has been confirmed to be an influencing factor by many researchers. Second, data screening was reasonable and the results obtained were reliable. Finally, the assessment of the nomogram and its ability to predict patient drug sensitivity provided improved clinical applicability.

Although the model we constructed presents the aforementioned advantages, there are also some shortcomings. For example, studies have shown seemingly contradictory results, the low-risk group has a higher degree of immune infiltration and a better prognosis, but the effects due to immunotherapy are poor. After reviewing the findings from study and referring to relevant prior studies, we presume that there may be several possible reasons for this result. First, we screened differentially expressed glycoly-related genes using the level of TMB. In the gene expression heatmap, we could find that the higher expression of the four genes in the model were all associated with a high TMB. The risk score was positively correlated with these four genes. Therefore, high TMB is also positively correlated with risk score. Current studies show that high TMB is positively correlated with the therapeutic effects of immune checkpoint inhibitors (Rizvi et al., 2015; Dong et al., 2017). Therefore, we believe that the results in this paper are reasonable, for the following reasons: 1) although the low-risk group showed greater immune cell infiltration, the cells that can directly affect immunotherapy are effector T cells; but in the microenvironment other immune cells can inhibit its function, thus making immunotherapy less effective (Cao et al., 2021). For example, this study showed that the low-risk group may have more macrophage M2 infiltration, as well as mast cells and neutrophils with dual roles. 2) The degree of influence of TMB on immunotherapy was higher than that of immune cell infiltration. The low-risk group was associated with a low TMB, and tumor cells with low TMB are not easily supervised by immune cells, even though there are more immune cells in the microenvironment, while a high TMB has a higher probability of triggering a T cell response (Jardim et al., 2021). Secondly, in previous studies, we also found that even though LUAD patients in the group achieved a superior response to immunotherapy, the actual patient survival was worse than the control group (Wang Q. et al., 2020), because the prognosis of tumor patients was influenced by multiple factors, including the sustainability of drug treatment, tolerability of adverse drug reactions, and response to radiotherapy. Moreover, it is a pity that we were not able to conduct *in vitro* studies to further verify these genes; thus, we can only extrapolate the function of these genes from existing studies. FKBP4 encodes a protein that plays a role in immune regulation and basic cellular processes are involved in protein folding and trafficking. Transcriptional activity and nuclear translocation of NF-κB is enhanced to promote LUAD progression through the formation of FKBP4/Hsp90/IKK and FKBP4/Hsp70/RelA complexes (Zong et al., 2021). As a target gene of mir-34A-5p, HMMR can promote tumor growth in LUAD through the HCG18/Mir-34A-5p/HMMR axis (Li et al., 2020c). As one of the beta-1,4-galactosyltransferase (beta4GalT) genes, B4GALT1 encodes an enzyme involved in glycoconjugates and lactose biosynthesis. Prior studies have found that it is associated with the prognosis of LUAD (Zhang et al., 2019), but the specific mechanisms involved have not been elucidated. SLC2A1 encodes a glucose transfer protein, which has been found to be associated with the prognosis of LUAD patients (Cheng et al., 2021), but the specific influencing mechanism has not been elaborated. STC1 can enhance glucose metabolism, ATP production, and lactic acid production of lung cancer cells under normoxic and hypoxic conditions, thus achieving anti-apoptotic properties (Ohkouchi et al., 2012). These genes deserve further investigation into the mechanisms and interactions of LUAD when experimental research resources become available in the future.

## Conclusion

An LUAD risk prognostic signature consisting of 5 glycolysis-related genes was identified in this study. To predict the survival time of patients with LUAD and their potential response to therapeutics, the model obtained in this study has excellent performance. This is the first study to predict the survival and drug response of patients with LUAD by combining glycolysis-related genes with TMB. Furthermore, the combination of glycolysis-related genes and immune responses not only enhances the accuracy of the model but also leads to a new direction in immunotherapy.

## Data Availability

The datasets presented in this study can be found in online repositories. The names of the repository/repositories and accession number(s) can be found below: The Cancer Genome Atlas, Gene Expression Omnibus (GEO), accession numbers GSE68465 and GSE11969.

## References

[B1] Bade B. C. Dela Cruz C. S. (2020). Lung Cancer 2020. Clin. Chest Med. 41 (1), 1–24. 10.1016/j.ccm.2019.10.001 32008623

[B2] Balachandran V. P. Gonen M. Smith J. J. DeMatteo R. P. (2015). Nomograms in Oncology: More Than Meets the Eye. Lancet Oncol. 16 (4), e173–e180. 10.1016/s1470-2045(14)71116-7 25846097 PMC4465353

[B3] Barta J. A. Powell C. A. Wisnivesky J. P. (2019). Global Epidemiology of Lung Cancer. Ann. Glob. Health 85 (1). 10.5334/aogh.2419 PMC6724220 30741509

[B4] Cancer Genome Atlas Research Network (2012). Comprehensive Genomic Characterization of Squamous Cell Lung Cancers. Nature 489(7417), 519–525. 10.1038/nature11404 22960745 PMC3466113

[B5] Cao R. Yuan L. Ma B. Wang G. Tian Y. (2021). Tumour Microenvironment (TME) Characterization Identified Prognosis and Immunotherapy Response in Muscle-Invasive Bladder Cancer (MIBC). Cancer Immunol. Immunother. 70 (1), 1–18. 10.1007/s00262-020-02649-x 32617668 PMC10973753

[B6] Cheng W.-C. Chang C.-Y. Lo C.-C. Hsieh C.-Y. Kuo T.-T. Tseng G.-C. (2021). Identification of Theranostic Factors for Patients Developing Metastasis after Surgery for Early-Stage Lung Adenocarcinoma. Theranostics 11 (8), 3661–3675. 10.7150/thno.53176 33664854 PMC7914355

[B7] Coy J. F. Dressler D. Wilde J. Schubert P. (2005). Mutations in the Transketolase-like Gene TKTL1: Clinical Implications for Neurodegenerative Diseases, Diabetes and Cancer. Clin. Lab. 51 (5-6), 257–273. 15991799

[B8] Danial N. N. Gramm C. F. Scorrano L. Zhang C.-Y. Krauss S. Ranger A. M. (2003). BAD and Glucokinase Reside in a Mitochondrial Complex that Integrates Glycolysis and Apoptosis. Nature 424 (6951), 952–956. 10.1038/nature01825 12931191

[B9] Dong Z.-Y. Zhong W.-Z. Zhang X.-C. Su J. Xie Z. Liu S.-Y. (2017). Potential Predictive Value of TP53 and KRAS Mutation Status for Response to PD-1 Blockade Immunotherapy in Lung Adenocarcinoma. Clin. Cancer Res. 23 (12), 3012–3024. 10.1158/1078-0432.Ccr-16-2554 28039262

[B10] Gao X. Tang M. Tian S. Li J. Liu W. (2021). A Ferroptosis-Related Gene Signature Predicts Overall Survival in Patients with Lung Adenocarcinoma. Future Oncol. 17 (12), 1533–1544. 10.2217/fon-2020-1113 33432837

[B11] Herbst R. S. Giaccone G. de Marinis F. Reinmuth N. Vergnenegre A. Barrios C. H. (2020). Atezolizumab for First-Line Treatment of PD-L1-Selected Patients with NSCLC. N. Engl. J. Med. 383 (14), 1328–1339. 10.1056/NEJMoa1917346 32997907

[B12] Hoshida Y. Brunet J.-P. Tamayo P. Golub T. R. Mesirov J. P. (2007). Subclass Mapping: Identifying Common Subtypes in Independent Disease Data Sets. PLoS One 2 (11), e1195. 10.1371/journal.pone.0001195 18030330 PMC2065909

[B13] Jardim D. L. Goodman A. de Melo Gagliato D. Kurzrock R. (2021). The Challenges of Tumor Mutational Burden as an Immunotherapy Biomarker. Cancer Cell 39 (2), 154–173. 10.1016/j.ccell.2020.10.001 33125859 PMC7878292

[B14] Jia S. Li L. Xie L. Zhang W. Zhu T. Qian B. (2021). Transcriptome Based Estrogen Related Genes Biomarkers for Diagnosis and Prognosis in Non-small Cell Lung Cancer. Front. Genet. 12, 666396. 10.3389/fgene.2021.666396 33936178 PMC8081391

[B15] Jiang P. Gu S. Pan D. Fu J. Sahu A. Hu X. (2018). Signatures of T Cell Dysfunction and Exclusion Predict Cancer Immunotherapy Response. Nat. Med. 24 (10), 1550–1558. 10.1038/s41591-018-0136-1 30127393 PMC6487502

[B16] Kang J. Zhang X.-C. Chen H.-J. Zhong W.-Z. Xu Y. Su J. (2020b). Complex ALK Fusions Are Associated with Better Prognosis in Advanced Non-small Cell Lung Cancer. Front. Oncol. 10, 596937. 10.3389/fonc.2020.596937 33363027 PMC7759679

[B17] Kang K. Xie F. Mao J. Bai Y. Wang X. (2020a). Significance of Tumor Mutation Burden in Immune Infiltration and Prognosis in Cutaneous Melanoma. Front. Oncol. 10, 573141. 10.3389/fonc.2020.573141 33072607 PMC7531222

[B18] Khan M. Lin J. Liao G. Tian Y. Liang Y. Li R. (2018). ALK Inhibitors in the Treatment of ALK Positive NSCLC. Front. Oncol. 8, 557. 10.3389/fonc.2018.00557 30687633 PMC6333640

[B19] Koppenol W. H. Bounds P. L. Dang C. V. (2011). Otto Warburg's Contributions to Current Concepts of Cancer Metabolism. Nat. Rev. Cancer 11 (5), 325–337. 10.1038/nrc3038 21508971

[B20] Li J. Wang H. Li Z. Zhang C. Zhang C. Li C. (2020a). A 5-Gene Signature Is Closely Related to Tumor Immune Microenvironment and Predicts the Prognosis of Patients with Non-small Cell Lung Cancer. BioMed Res. Int. 2020, 1–9. 10.1155/2020/2147397 PMC6975218 31998783

[B21] Li W. Gao L.-N. Song P.-P. You C.-G. (2020b). Development and Validation of a RNA Binding Protein-Associated Prognostic Model for Lung Adenocarcinoma. Aging 12 (4), 3558–3573. 10.18632/aging.102828 32087603 PMC7066909

[B22] Li W. Pan T. Jiang W. Zhao H. (2020c). HCG18/miR-34a-5p/HMMR axis Accelerates the Progression of Lung Adenocarcinoma. Biomed. Pharmacother. 129, 110217. 10.1016/j.biopha.2020.110217 32559619

[B23] Liu J. Li S. Feng G. Meng H. Nie S. Sun R. (2020). Nine Glycolysis-Related Gene Signature Predicting the Survival of Patients with Endometrial Adenocarcinoma. Cancer Cell Int. 20, 183. 10.1186/s12935-020-01264-1 32489319 PMC7247270

[B24] Liu Z. Guo C. Dang Q. Wang L. Liu L. Weng S. (2022a). Integrative Analysis from Multi-Center Studies Identities a Consensus Machine Learning-Derived lncRNA Signature for Stage II/III Colorectal Cancer. EBioMedicine 75, 103750. 10.1016/j.ebiom.2021.103750 34922323 PMC8686027

[B25] Liu Z. Liu L. Weng S. Guo C. Dang Q. Xu H. (2022b). Machine Learning-Based Integration Develops an Immune-Derived lncRNA Signature for Improving Outcomes in Colorectal Cancer. Nat. Commun. 13 (1), 816. 10.1038/s41467-022-28421-6 35145098 PMC8831564

[B26] Liu Z. Lu T. Li J. Wang L. Xu K. Dang Q. (2021). Development and Clinical Validation of a Novel Six-Gene Signature for Accurately Predicting the Recurrence Risk of Patients with Stage II/III Colorectal Cancer. Cancer Cell Int. 21 (1), 359. 10.1186/s12935-021-02070-z 34233675 PMC8265123

[B27] Liu Z. Xu H. Weng S. Ren Y. Han X. (2022c). Stemness Refines the Classification of Colorectal Cancer with Stratified Prognosis, Multi-Omics Landscape, Potential Mechanisms, and Treatment Options. Front. Immunol. 13, 828330. 10.3389/fimmu.2022.828330 35154148 PMC8828967

[B28] Luo C. Lei M. Zhang Y. Zhang Q. Li L. Lian J. (2020). Systematic Construction and Validation of an Immune Prognostic Model for Lung Adenocarcinoma. J. Cell Mol. Med. 24 (2), 1233–1244. 10.1111/jcmm.14719 31779055 PMC6991688

[B29] Lv J. Zhu Y. Ji A. Zhang Q. Liao G. (2020). Mining TCGA Database for Tumor Mutation Burden and Their Clinical Significance in Bladder Cancer. Biosci. Rep. 40 (4). 10.1042/bsr20194337 PMC7178217 32239176

[B30] Ohkouchi S. Block G. J. Katsha A. M. Kanehira M. Ebina M. Kikuchi T. (2012). Mesenchymal Stromal Cells Protect Cancer Cells from ROS-Induced Apoptosis and Enhance the Warburg Effect by Secreting STC1. Mol. Ther. 20 (2), 417–423. 10.1038/mt.2011.259 22146344 PMC3277221

[B31] Pelicano H. Martin D. S. Xu R.-H. Huang P. (2006). Glycolysis Inhibition for Anticancer Treatment. Oncogene 25 (34), 4633–4646. 10.1038/sj.onc.1209597 16892078

[B32] Rizvi N. A. Hellmann M. D. Snyder A. Kvistborg P. Makarov V. Havel J. J. (2015). Mutational Landscape Determines Sensitivity to PD-1 Blockade in Non-small Cell Lung Cancer. Science 348 (6230), 124–128. 10.1126/science.aaa1348 25765070 PMC4993154

[B33] Schwartz L. Supuran C. Alfarouk K. (2017). The Warburg Effect and the Hallmarks of Cancer. Acamc 17 (2), 164–170. 10.2174/1871520616666161031143301 27804847

[B34] Shan L. Wang Z. Guo L. Sun H. Qiu T. Ling Y. (2015). Concurrence of EGFR Amplification and Sensitizing Mutations Indicate a Better Survival Benefit from EGFR-TKI Therapy in Lung Adenocarcinoma Patients. Lung Cancer 89 (3), 337–342. 10.1016/j.lungcan.2015.06.008 26141217

[B35] Snyder A. Makarov V. Merghoub T. Yuan J. Zaretsky J. M. Desrichard A. (2014). Genetic Basis for Clinical Response to CTLA-4 Blockade in Melanoma. N. Engl. J. Med. 371 (23), 2189–2199. 10.1056/NEJMoa1406498 25409260 PMC4315319

[B36] Sparano J. A. Gray R. J. Makower D. F. Pritchard K. I. Albain K. S. Hayes D. F. (2018). Adjuvant Chemotherapy Guided by a 21-Gene Expression Assay in Breast Cancer. N. Engl. J. Med. 379 (2), 111–121. 10.1056/NEJMoa1804710 29860917 PMC6172658

[B37] Sun S. Guo W. Wang Z. Wang X. Zhang G. Zhang H. (2020). Development and Validation of an Immune‐related Prognostic Signature in Lung Adenocarcinoma. Cancer Med. 9 (16), 5960–5975. 10.1002/cam4.3240 32592319 PMC7433810

[B38] Tang J. Luo Y. Wu G. (2020). A Glycolysis-Related Gene Expression Signature in Predicting Recurrence of Breast Cancer. Aging 12 (24), 24983–24994. 10.18632/aging.103806 33201835 PMC7803557

[B39] Travis W. D. Brambilla E. Burke A. P. Marx A. Nicholson A. G. (2015). Introduction to the 2015 World Health Organization Classification of Tumors of the Lung, Pleura, Thymus, and Heart. J. Thorac. Oncol. 10 (9), 1240–1242. 10.1097/jto.0000000000000663 26291007

[B40] Vaupel P. Schmidberger H. Mayer A. (2019). The Warburg Effect: Essential Part of Metabolic Reprogramming and Central Contributor to Cancer Progression. Int. J. Radiat. Biol. 95 (7), 912–919. 10.1080/09553002.2019.1589653 30822194

[B41] Vogelstein B. Papadopoulos N. Velculescu V. E. Zhou S. Diaz L. A. Jr. Kinzler K. W. (2013). Cancer Genome Landscapes. Science 339 (6127), 1546–1558. 10.1126/science.1235122 23539594 PMC3749880

[B42] Wang Q. Li M. Yang M. Yang Y. Song F. Zhang W. (2020b). Analysis of Immune-Related Signatures of Lung Adenocarcinoma Identified Two Distinct Subtypes: Implications for Immune Checkpoint Blockade Therapy. Aging 12 (4), 3312–3339. 10.18632/aging.102814 32091408 PMC7066911

[B43] Wang X. Yao S. Xiao Z. Gong J. Liu Z. Han B. (2020a). Development and Validation of a Survival Model for Lung Adenocarcinoma Based on Autophagy-Associated Genes. J. Transl. Med. 18 (1), 149. 10.1186/s12967-020-02321-z 32238163 PMC7115085

[B44] Wu C. Cai X. Yan J. Deng A. Cao Y. Zhu X. (2020). Identification of Novel Glycolysis-Related Gene Signatures Associated with Prognosis of Patients with Clear Cell Renal Cell Carcinoma Based on TCGA. Front. Genet. 11, 589663. 10.3389/fgene.2020.589663 33391344 PMC7775602

[B45] Wu L. Wen Z. Song Y. Wang L. (2021). A Novel Autophagy‐related lncRNA Survival Model for Lung Adenocarcinoma. J. Cell Mol. Med. 25 (12), 5681–5690. 10.1111/jcmm.16582 33987935 PMC8184679

[B46] Xu R. H. Pelicano H. Zhou Y. Carew J. S. Feng L. Bhalla K. N. (2005). Inhibition of Glycolysis in Cancer Cells: a Novel Strategy to Overcome Drug Resistance Associated with Mitochondrial Respiratory Defect and Hypoxia. Cancer Res. 65 (2), 613–621. 15695406

[B47] Xu X. D. Shao S. X. Jiang H. P. Cao Y. W. Wang Y. H. Yang X. C. (2015). Warburg Effect or Reverse Warburg Effect? A Review of Cancer Metabolism. Oncol. Res. Treat. 38 (3), 117–122. 10.1159/000375435 25792083

[B48] Xu Z.-y. Zhao M. Chen W. Li K. Qin F. Xiang W.-w. (2020). Analysis of Prognostic Genes in the Tumor Microenvironment of Lung Adenocarcinoma. PeerJ 8, e9530. 10.7717/peerj.9530 32775050 PMC7382940

[B49] Yang J. Ren B. Yang G. Wang H. Chen G. You L. (2020). The Enhancement of Glycolysis Regulates Pancreatic Cancer Metastasis. Cell. Mol. Life Sci. 77 (2), 305–321. 10.1007/s00018-019-03278-z 31432232 PMC11104916

[B50] Yao Y. Zhang T. Qi L. Liu R. Liu G. Li J. (2021). Identification of Four Genes as Prognosis Signatures in Lung Adenocarcinoma Microenvironment. Pgpm 14, 15–26. 10.2147/pgpm.S283414 33447073 PMC7802904

[B51] Yu Y. Tian X. (2020). Analysis of Genes Associated with Prognosis of Lung Adenocarcinoma Based on GEO and TCGA Databases. Medicine 99 (19), e20183. 10.1097/md.0000000000020183 32384511 PMC7220259

[B52] Yue C. Ma H. Zhou Y. (2019). Identification of Prognostic Gene Signature Associated with Microenvironment of Lung Adenocarcinoma. PeerJ 7, e8128. 10.7717/peerj.8128 31803536 PMC6886493

[B53] Zhang L. Li B. Peng Y. Wu F. Li Q. Lin Z. (2020). The Prognostic Value of TMB and the Relationship between TMB and Immune Infiltration in Head and Neck Squamous Cell Carcinoma: A Gene Expression-Based Study. Oral Oncol. 110, 104943. 10.1016/j.oraloncology.2020.104943 32919362

[B54] Zhang L. Zhang Z. Yu Z. (2019). Identification of a Novel Glycolysis-Related Gene Signature for Predicting Metastasis and Survival in Patients with Lung Adenocarcinoma. J. Transl. Med. 17 (1), 423. 10.1186/s12967-019-02173-2 31847905 PMC6916245

[B55] Zheng Q. Min S. Zhou Q. (2021). Identification of Potential Diagnostic and Prognostic Biomarkers for LUAD Based on TCGA and GEO Databases. Biosci. Rep. 41 (6). 10.1042/bsr20204370 PMC8182989 34017995

[B56] Zhou W. Zhang S. Cai Z. Gao F. Deng W. Wen Y. (2020). A Glycolysis-Related Gene Pairs Signature Predicts Prognosis in Patients with Hepatocellular Carcinoma. PeerJ 8, e9944. 10.7717/peerj.9944 33062428 PMC7531359

[B57] Zhu J.-q. Zhong W.-z. Zhang G.-c. Li R. Zhang X.-c. Guo A.-l. (2008). Better Survival with EGFR Exon 19 Than Exon 21 Mutations in Gefitinib-Treated Non-small Cell Lung Cancer Patients Is Due to Differential Inhibition of Downstream Signals. Cancer Lett. 265 (2), 307–317. 10.1016/j.canlet.2008.02.064 18407408

[B58] Zhu K. Liu Q. Zhou Y. Tao C. Zhao Z. Sun J. (2015). Oncogenes and Tumor Suppressor Genes: Comparative Genomics and Network Perspectives. BMC Genomics 16 (Suppl. 7Suppl 7). 10.1186/1471-2164-16-s7-s8 PMC4474543 26099335

[B59] Zong S. Jiao Y. Liu X. Mu W. Yuan X. Qu Y. (2021). FKBP4 Integrates FKBP4/Hsp90/IKK with FKBP4/Hsp70/RelA Complex to Promote Lung Adenocarcinoma Progression via IKK/NF-κB Signaling. Cell Death Dis. 12 (6), 602. 10.1038/s41419-021-03857-8 34112753 PMC8192522

