# Supplementary material for: Predicting Differences in Treatment Response and Survival Time of Lung Adenocarcinoma Patients Based on a Prognostic Risk Model of Glycolysis-Related Genes
Source: Front Genet. 2022 May 25;13:828543. doi: 10.3389/fgene.2022.828543 (PMC9174756; doi:10.3389/fgene.2022.828543)
Supplement: Supplementary file 1 [file Table1.doc]

**Supplementary Table 1** Multivariate Cox proportional hazards regression coefficient

| Gene | 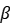 |
| --- | --- |
| FKBP4 | 0.00565 |
| HMMR | 0.03539 |
| B4GALT1 | 0.00638 |
| SLC2A1 | 0.00332 |
| STC1 | 0.00387 |
