# Supplementary figures and images for: Predicting Differences in Treatment Response and Survival Time of Lung Adenocarcinoma Patients Based on a Prognostic Risk Model of Glycolysis-Related Genes
Source: Front Genet. 2022 May 25;13:828543. doi: 10.3389/fgene.2022.828543 (PMC9174756; doi:10.3389/fgene.2022.828543)

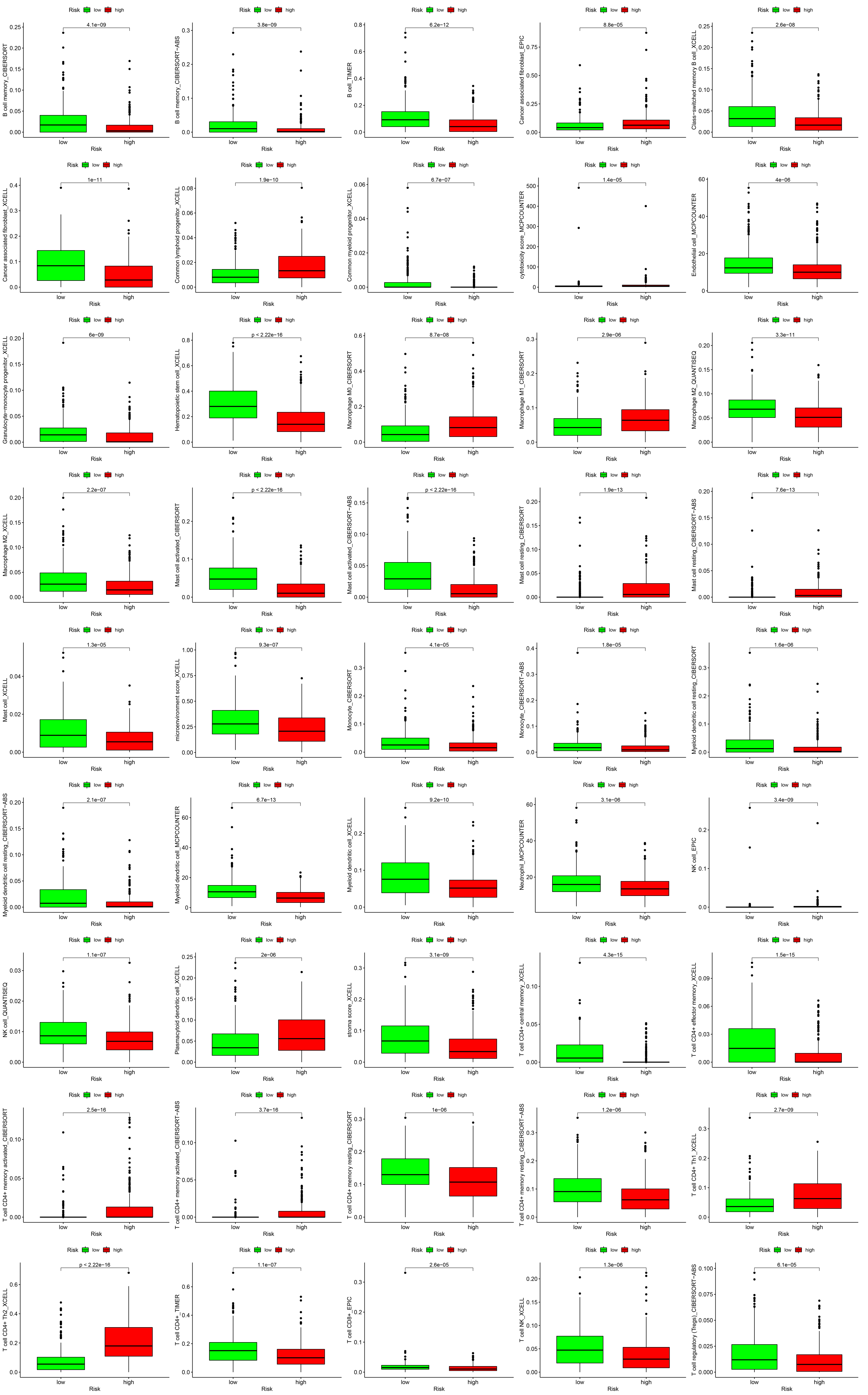

Supplement: Supplementary file 2 [file DataSheet1.PDF]
